# Hypoxia Transiently Sequesters Mps1 and Polo to Collagenase-Sensitive Filaments in *Drosophila* Prometaphase Oocytes

**DOI:** 10.1371/journal.pone.0007544

**Published:** 2009-10-22

**Authors:** William D. Gilliland, Dana L. Vietti, Nicole M. Schweppe, Fengli Guo, Teri J. Johnson, R. Scott Hawley

**Affiliations:** 1 Stowers Institute for Medical Research, Kansas City, Missouri, United States of America; 2 Department of Biological Sciences, DePaul University, Chicago, Illinois, United States of America; 3 University of Kansas Medical Center, Kansas City, Kansas, United States of America; 4 Kansas City University of Medicine and Biosciences, Kansas City, Missouri, United States of America; 5 Department of Physiology, University of Kansas Medical Center, Kansas City, Kansas, United States of America; 6 American Cancer Society Research Professor, Atlanta, Georgia, United States of America; University of Birmingham, United Kingdom

## Abstract

**Background:**

The protein kinases Mps1 and Polo, which are required for proper cell cycle regulation in meiosis and mitosis, localize to numerous ooplasmic filaments during prometaphase in *Drosophila* oocytes. These filaments first appear throughout the oocyte at the end of prophase and are disassembled after egg activation.

**Methodology/Principal Findings:**

We showed here that Mps1 and Polo proteins undergo dynamic and reversible localization to static ooplasmic filaments as part of an oocyte-specific response to hypoxia. The observation that Mps1- and Polo-associated filaments reappear in the same locations through multiple cycles of oxygen deprivation demonstrates that underlying structural components of the filaments must still be present during normoxic conditions. Using immuno-electron microscopy, we observed triple-helical binding of Mps1 to numerous electron-dense filaments, with the gold label wrapped around the outside of the filaments like a garland. In addition, we showed that in live oocytes the relocalization of Mps1 and Polo to filaments is sensitive to injection of collagenase, suggesting that the structural components of the filaments are composed of collagen-like fibrils. However, the collagen-like genes we have been able to test so far (*vkg* and *CG42453*) did not appear to be associated with the filaments, demonstrating that the collagenase-sensitive component of the filaments is one of a number of other *Drosophila* proteins bearing a collagenase cleavage site. Finally, as hypoxia is known to cause Mps1 protein to accumulate at kinetochores in syncytial embryos, we also show that GFP-Polo accumulates at both kinetochores and centrosomes in hypoxic syncytial embryos.

**Conclusions/Significance:**

These findings identify both a novel cellular structure (the ooplasmic filaments) as well as a new localization pattern for Mps1 and Polo and demonstrate that hypoxia affects Polo localization in *Drosophila*.

## Introduction

The *Drosophila* ovary has proven to be a useful model system for studying the mechanisms by which the processes of oocyte maturation and chromosome segregation interact with or are controlled by the meiotic cell cycle. The ovary is organized as a bundle of individual ovarioles, each of which contains germline stem cells at its anterior tip. These stem cells give rise to 16-cell cysts that mature into functional oocytes as the cysts move along the ovariole. After pre-meiotic DNA replication and recombination are completed, the oocyte enters an extended prophase while the cyst matures, concluding with nuclear envelope breakdown (NEB). After NEB, the meiotic chromosomes build an acentriolar spindle [1], and then undergo an extended prometaphase that concludes with the chromosomes arresting at metaphase I [2].

In addition to the changes in the oocyte nucleus, numerous changes in the 16-cell cyst and its contents are also taking place throughout oocyte development, including the growth and degradation of the polytene nurse and follicle cells [3], the kenotic dumping of nurse cell contents into the oocyte [4], the formation of a membranous sheath around the meiotic spindle [5], the growth of the dorsal appendages [6], and the maturation of the vitelline membrane and chorion [7]. These processes produce the phenotypic landmarks that are used to divide oogenesis into 14 stages [8], [9]. Keeping the meiotic cell cycle entrained to the status of oogenesis requires the activity of a number of cell cycle regulatory proteins, including the activities of cyclins [10], [11], the Cdc25 homolog Twine [12], the spindle assembly checkpoint protein Ald/Mps1 (hereafter referred to as Mps1) [13], [14] and Polo kinase [15].

Although Polo and Mps1 are known to be localized at kinetochores in many organisms and cell types, including *Drosophila* oocytes [16], [17], [18], [19], [20], we have previously shown that in *Drosophila* prometaphase oocytes both Polo and Mps1 colocalize to numerous filaments throughout the ooplasm [20]. These filaments, which were present in ∼70% of wildtype oocytes, did not colocalize with tested candidate structural proteins (including tubulin, actin, anillin, septin, or lamin), appeared to polymerize at the onset of NEB [20], and were not observed in syncytial embryos, although some early preblastoderm embryos showed Mps1 localization to small filaments or foci [21], consistent with the filaments being disassembled shortly after fertilization. These filaments have also not been observed in somatic cells, such as in colchicine-treated larval brain squashes [20]. Therefore, the assembly and disassembly of these filaments appears to be taking place in parallel with the maturation of other oocyte contents at NEB, and the localization of Mps1 and Polo to the filaments could potentially have important functional consequences for the regulation of this process. Interestingly, despite the fact that there is no Mps1 homolog in the nematode *C. elegans* [22], similar-appearing filaments containing kinetochore components in *C. elegans* prometaphase oocytes have also been reported [23], suggesting that these filaments may represent an evolutionarily conserved structure.

While our previous study reported the existence of these filaments in *Drosophila* oocytes, it did not identify the structural backbone components, address their functional role in the oocyte, or determine why they were not present in all oocytes. Here, we demonstrate that rather than being components of the filaments themselves, Mps1 and Polo proteins are transiently localized to the filaments in response to hypoxia on a time scale of approximately 10 minutes. This localization is reversible, and repeated exposure to hypoxia indicates that the filaments are static structures to which Mps1 and Polo become sequestered. Furthermore, we use immunogold electron microscopy to characterize the filaments as being proteinaceous structures approximately 150 nm in diameter, with the Mps1 localizing in a triple-helical pattern on the surface. The Mps1 and Polo localization to filaments is also disrupted by the injection of collagenase, suggesting that the structural components of the filaments include one of a number of proteins bearing a collagenase cleavage site in the *Drosophila* genome. However, the two collagen-like genes we have tested (*vkg* and *CG42543*) have not appeared to associate with the filaments. Finally, similar to Mps1 [21], we show that during mitosis hypoxia changes the localization of Polo, suggesting a role for this protein in mediating the hypoxic response.

## Results

### Localization of Mps1 and Polo to filaments is a transient response to hypoxia

**Figure 1:**
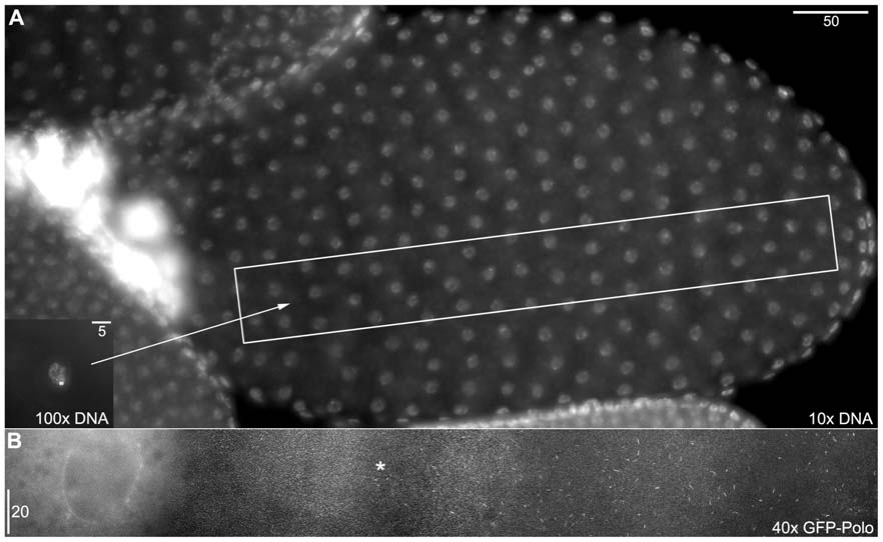
GFP-Polo Oocyte fixed in mid-NEB with nascent filament formation. 1A. An image of a late stage 12 or early stage 13 GFP-Polo oocyte, which was presumably fixed in the early stages of nuclear envelope breakdown (NEB), with the anterior end of the oocyte on the left and the posterior end on the right. While this image is of a fixed sample and it is therefore impossible to be certain that this oocyte is undergoing NEB, the characteristics of the cell (the shape of the karyosome (inset), the growth of the dorsal appendages, and the appearance of the nuclear envelope) are all similar to those of oocytes that were imaged going through NEB. The GFP-Polo filaments cannot be seen at this magnification. 1B. A composite of five individual image stacks, showing the GFP-Polo localization of the region inside the rectangle in 1A. Note the GFP-Polo localization to the nuclear envelope on the left. The filaments become shorter and less abundant moving across the oocyte from posterior to anterior, with the location of the last resolvable filaments indicated (asterisk). This pattern is consistent with the linear polymerization of filaments occurring at NEB, with filaments growing linearly from “seeds” and polymerization being triggered by a wave that propagates across the oocyte from posterior to anterior.

Based on fixed images, we reported that Mps1 and Polo filament formation appeared to initiate at the onset of nuclear envelope breakdown (NEB) [20], an observation that has been confirmed by analysis of subsequent oocytes that were fixed during the process of NEB (Figure 1). Our analysis of this and other oocytes undergoing NEB (data not shown) suggested that filament formation is propagating in a wave from the posterior to anterior end of the oocyte, with filaments growing and becoming more numerous as NEB proceeds. Using our standard protocol for long-duration live-imaging [24], we examined living oocytes expressing either GFP-Mps1 [16] or GFP-Polo [25] that would be expected to have completed NEB based on dorsal appendage development [2]. However, using this protocol we were unable to observe GFP-Mps1 or GFP-Polo localized to filaments in living oocytes, with fluorescence being instead distributed evenly across the ooplasm.

This failure to observe oocytes in long-duration live-imaging was unexpected, as approximately 70% of fixed prometaphase oocytes contained filaments, and our previous study had readily observed GFP filaments with both fixed oocytes expressing GFP-Mps1 and in live oocytes expressing GFP-Polo. This was also not an artifact of the GFP tag, as filaments were first identified by antibodies against native Mps1 protein, and we had previously demonstrated that GFP-Polo and anti-Mps1 antibody highlighted the same structures [20]. The previous observation of GFP-Polo filaments in live oocytes also ruled out the possibility that the failure to find filaments was a fixation artifact, and indicated that the cause of the failure to find the filaments using our standard protocol for live-imaging reflected a way the experiment was methodologically different from previous GFP imaging experiments.

Among the methodological differences between the previously published live imaging of the GFP-Polo line and our standard long duration live-imaging protocol were the steps taken to prolong oocyte viability. When preparing oocytes for GFP-Polo imaging, females had been anesthetized with CO_2_ and dissected oocytes were covered with a glass coverslip for immediate visualization. However, using our standard protocol for long duration live-imaging analysis of oocytes [24] preparations are covered in an oxygen-permeable membrane, and the time required for sample preparation meant that imaging did not begin until ∼20 minutes after anesthetization. As Mps1 is required to correctly arrest mitotic *Drosophila* cells in response to hypoxia [16] and is relocalized to kinetochores during hypoxia in mitotic cells [21], we hypothesized that the localization of Mps1 to ooplasmic filaments was also a response to hypoxia. The previous GFP-Polo experiments would then have found GFP-Polo attached to filaments due to the oocytes still being hypoxic after CO_2_ anesthetization of the flies prior to fixation.

**Figure 2:**
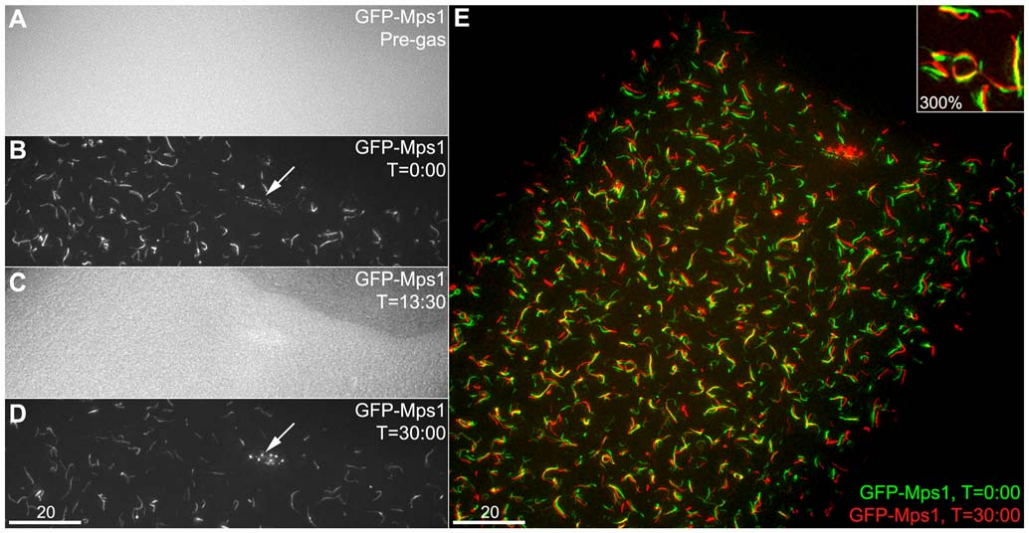
Hypoxia reversibly sequesters GFP-Mps1 to filaments. A live stage 13 GFP-Mps1 oocyte, positioned with the anterior end at the upper right and the posterior end towards the lower left, shows reversible sequestration of GFP-Mps1 to filaments under hypoxia. The entire cell was larger than the microscope's field of view at this magnification. Five other oocytes on the same slide were monitored (but not imaged) at the 2A, 2B, and 2D time points; all exhibited similar localization of GFP-Mps1 to filaments. 2A: Prior to exposure to CO_2_, the oocyte does not exhibit GFP-Mps1 localization to filaments. 2B: After exposure to CO_2_, GFP-Mps1 shows localization to both ooplasmic filaments as well as small foci along the meiotic spindle (arrow). This image was acquired just prior to the 0:00 time point in Movie S1. 2C: After restoration of ambient air, the GFP has diffused back into the ooplasm and the filaments are no longer visible. This image is the 13∶30 frame of Movie S1. 2D: After reintroduction of CO_2_, GFP-Mps1 localization to filaments and the spindle has reappeared. Note that now the spindle localization (arrow) is in closely arranged bright foci, corresponding to the kinetochores of the meiotic chromosomes. This image was acquired just after the 30:00 time point in Movie S1. 2E: The before and after images from 2B and 2D are superimposed on each other as green (before) and red (after), respectively. This image shows that the cell has flattened out slightly, as the lateral width across the cell has increased, and some filaments have moved slightly. However, most of the filaments have reappeared with the same topology and in roughly the same locations (300% enlargement of area near center, inset).

To test this possibility, we prepared live oocytes with an oxygen permeable membrane, and after examination of oocytes to verify the absence of GFP filaments (Figure 2A), we induced hypoxia by filling a chamber covering the stage with CO_2_. For both GFP-Mps1 and GFP-Polo, GFP became visibly associated with filaments after approximately 8–12 minutes of CO_2_ (GFP-Mps1 shown in Figure 2B; GFP-Polo data not shown). After restoring ambient air, the GFP dispersed back into the ooplasm (Figure 2C, Movie S1) in approximately the same time required for the initial localization. Reapplication of CO_2_ restored the localization to filaments (Figure 2D), which despite some movement during the course of live-imaging, reappeared in approximately the same locations throughout the oocyte (Figure 2E).

Several lines of evidence indicate that this localization is a specific response to hypoxia and not a generalized stress response or due to CO_2_ acidification of buffer during incubation. First, filaments could be observed in live imaging by the use of N_2_ gas as well as CO_2_, and were also observed in flies that were held in CO_2_ for 10 minutes prior to dissection and immediate fixation (data not shown). Second, incubation of oocytes in the presence of sodium azide, which inhibits mitochondrial respiration and can trigger the hypoxic response in mitotic *Drosophila* cells [21], causes localization of GFP-Mps1 to the filaments. *In vitro* incubation of oocytes in 0.03% sodium azide resulted in 92% (34/37) of fixed GFP-Mps1 oocytes displaying filaments, compared to 3% (1/33) of incubation-only control oocytes (Figure S1). Third, heat-shocking GFP-Mps1 or GFP-Polo oocytes by incubating them under normoxic conditions at 37°C did not induce localization of GFP to filaments even after 18 minutes of heat shock. This lack of localization was not due to an inability of heat-shocked oocytes to carry out the localization, as subsequent exposure of those same oocytes to CO_2_ at 37°C led to GFP filament localization (Movie S2). These experiments demonstrate that localization of GFP-Polo and GFP-Mps1 to filaments is a specific response to hypoxia that does not occur in response to other forms of cellular stress, such as CO_2_ acidification or heat shock. We furthermore conclude, as the GFP-decorated filaments reappear in the same locations (Figure 2E), that Mps1 and Polo must localize to static scaffolds that are still present under normoxic conditions. According to this view, Mps1 and Polo are not structural components of the filaments, but instead are transiently sequestered to the filaments during the hypoxic response.

### Characterization of filaments by immunogold electron microscopy

**Figure 3:**
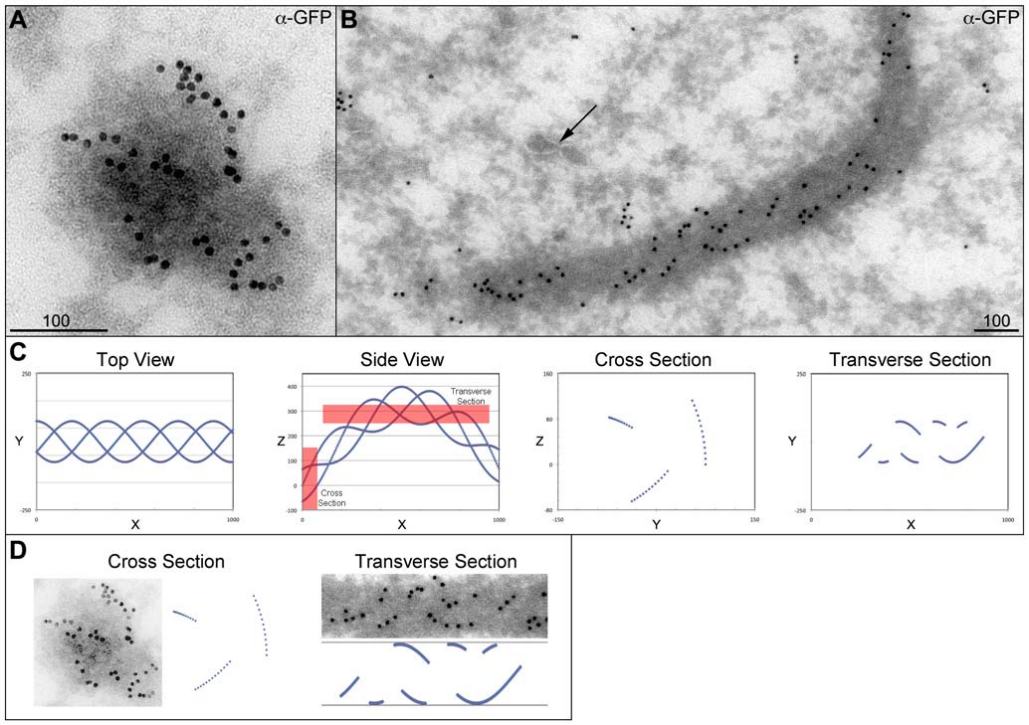
Immunogold localization of GFP-Mps1 to filaments. Hypoxic stage 13 GFP-Mps1 oocytes were examined by immuno-EM, using anti-GFP antibodies and 10 nm colloidal gold conjugated secondary antibodies. Sections were 70 nm thick, with scale bars equal to 100 nm. 3A: A cross section of a filament showing immunogold dots along the outside of the darker filament core. 3B: A transverse section of a filament, showing distinct rows of dots in a spiral pattern. As the filament begins and ends within this image, the filament must either be linear (entering and exiting the section through opposite sides) or curved (entering and exiting the section from the same side). Also note the presence of lipid membrane tracks (arrow), which do not appear to be associated with the filament. 3C: A triple helix was modeled as three lines moving around a bent cylinder (see Materials and Methods), using values for the radius and spiral length measured from the image in 3B, and simulated 70 nm cross and transverse sections of the structure were made (red boxes). Note that the EM sections are a three-dimensional volume “projected” along one axis (X for cross section, Z for transverse) onto a two-dimensional plane. The gaps in the transverse section correspond to the parts of the helix that travel outside the section. 3D: Side-by-side comparison of the cross sections and transverse sections from the EM images and the model. This shows that while the model is not an exact match, the model recapitulates many of the features of the actual data.

To better characterize the structural nature of these filaments, we set out to examine GFP-Mps1 decorated filaments by electron microscopy. We incubated GFP-Mps1 oocytes under conditions that were known to produce robust Mps1-bearing filaments. Oocytes were then fixed and those with strong GFP filaments were selected for postfixation, cryosectioning and labeling with anti-GFP antibody followed by visualization with gold-conjugated secondary antibody. The immunogold label was found to be associated with long protein-dense fibers of approximately 150 nm in diameter (Figure 3). These fibers are entirely consistent with those observed by fluorescent cytology, as this diameter would be well below the half-wavelength resolution limit of the light microscope and consistent with their appearance as thin fibers. In both cross sections (Figure 3A) and transverse sections (Figure 3B) of the fibers, the immunogold label is associated in rows along the surface of the fibers, which appears in a multiply helical configuration. The transverse filament is especially striking, and is consistent with a section through a “barber pole” helical structure (Figure 3C). These images also indicated that the filament structures did not appear to contain bilayer membranes, which could be seen in the background of the images (Figure 3B), but were not observed within or around the electron-dense filaments. This rules out the possibility of these filaments being mitochondria, golgi or ER-associated membrane tubules [26], [27], [28], [29].

### Localization of GFP to filaments is sensitive to collagenase

While the characterization of the proteinaceous filaments by immuno-EM revealed the gross morphology of the filaments, it left unanswered the question of the protein composition of the static filaments. The arrangement of the gold particles in some images suggested a triple helical structure, which led us to propose collagen, a structural protein that we had not previously considered as it is normally associated with the extracellular matrix. Collagen IV proteins are stockpiled in the developing oocyte [30], and while an individual collagen fiber would be much too narrow to match the diameter of the filaments observed by EM, fibrils formed from bundles of multiple collagen fibers can easily be as wide as 150 nm in diameter [31].

**Figure 4:**
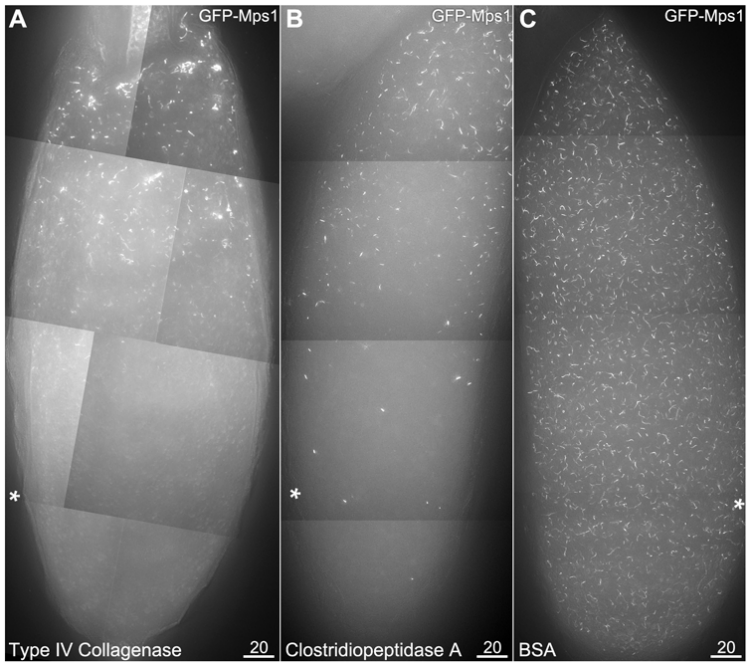
Injected collagenase enzyme disrupts localization to filaments. Oocytes are positioned with their anterior ends at the top, and injection sites are indicated by asterisks. As the oocyte is too large to be imaged in its entirety at this magnification, images are composites of multiple image stacks, acquired and combined using the Panels and Stitch functions in SoftWoRx. GFP-Mps1 is shown; GFP-Polo responded similarly (data not shown). 4A: Injection of a crude collagenase (Type IV, Gibco) prevented the localization of GFP-Mps1 to filaments in the region around the injection site under hypoxia. 4B: Injection of high purity Clostridiopeptidase A enzyme (Sigma-Aldrich) also prevented the localization of GFP-Mps1 to filaments after exposure to CO_2_ in the region surrounding the injection site (asterisk). 4C: Control injection of water (data not shown) or a protein solution (5 mg/ml BSA) did not disrupt localization of GFP-Mps1 to the filaments.

To test whether these filaments contained collagen, we injected GFP-Mps1 and GFP-Polo oocytes with collagenase enzyme, then oocytes were made hypoxic by incubation in CO_2_ and observed (GFP-Mps1 shown in Figure 4; GFP-Polo data not shown). For both a crude collagenase fraction (Figure 4A) and a purified enzyme (Figure 4B) the localization of GFP-Mps1 and GFP-Polo to filaments was eliminated around the injection site, with injection of concentrations as low as 100 µg/ml causing disruption. This disruption was not due to quenching of the GFP, as the diffuse fluorescence of unlocalized protein did not appear decreased. This was also not a consequence of the injection alone, as our previous injections of actin or tubulin poisons, or control injections of water or protein (5 mg/ml BSA), did not interfere with the localization to the filaments (Figure 4C). Therefore, one or more of the structural components of the filament backbone, or possibly the proteins required to transport Mps1 and Polo proteins to the filaments, must be susceptible to collagenase cleavage.

There are only three collagen genes conserved in the *Drosophila* genome, two type IV collagens *(Dcg1*, *vkg*) and one type XIV/XVIII *(CG42543)* [32]. We have tested a stock expressing Vkg-GFP [33], which did not show any localization in the ooplasm (data not shown). We also tested homozygotes for *CG42543^f07253^*, a homozygous viable allele caused by a Piggybac insertion into protein coding sequence [34]. Based on anti-Mps1 antibody localization [20], this allele did not appear to have any effect on Mps1 localization to filaments during hypoxia (data not shown). Searching the annotated protein sequences of *D. melanogaster* for the collagenase (Clostridiopeptidase A) cleavage site Pro-X-Gly-Pro, where X is any neutral amino acid [35], identifies 915 proteins that contain one or more such sequences, including all three annotated *Drosophila* collagens.

### Polo localization in mitosis is sensitive to hypoxia

**Figure 5:**
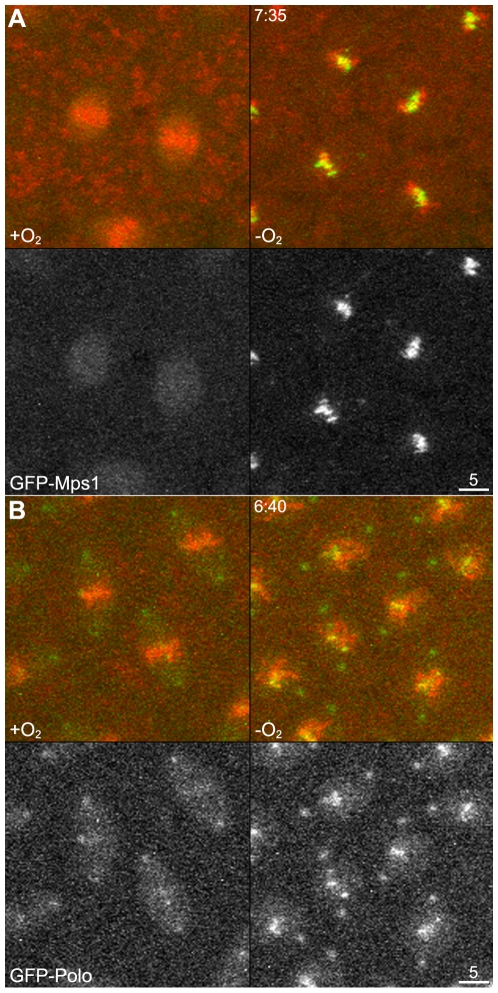
Hypoxia changes localization of Polo during mitosis. Metaphase chromosomes in embryos expressing either GFP-Mps1 or GFP-Polo (green) and a red fluorescent histone (His2AvD-tDimer, red) are presented. Each is shown at two successive cycles of mitosis, with the central two nuclei in the first cycle (left) dividing vertically to give rise to the central four nuclei in the next cycle (right). The left cycle is normoxic, and embryos underwent hypoxic arrest in metaphase of the next cell cycle after the flow of CO_2_ was turned on. Both merged and GFP-only signals are shown. Timestamps are the time elapsed since the CO_2_ was turned on. Both embryos resumed mitotic cycling after a return to normoxia (GFP-Mps1 data not shown; GFP-Polo in Movie S3). 5A: An embryo expressing GFP-Mps1 shows diffuse Mps1 signal around the chromosomes during normoxia (left), and kinetochore localization during hypoxia (right). This demonstrates that we can recreate the published localization of Mps1 in response to hypoxia [21] using our apparatus. 5B: The same embryo as shown in Movie S3, at metaphase of successive cell cycles, showing that GFP-Polo localizes to centrosomes, spindle and kinetochores during normoxia, while during hypoxia it is enriched only at centrosomes and kinetochores.

Hypoxia causes mitosis to rapidly arrest in syncytial *Drosophila* oocytes [36], and during that arrest Mps1 strongly localizes to metaphase kinetochores [21]. As hypoxia causes Mps1 and Polo to relocalize to filaments in oocytes, we determined if hypoxia also causes Polo to localize in mitotic cells. Using embryos containing GFP-Mps1 and a red fluorescent histone conjugate (His2AvD-tDimer [37]) we were able to reproduce the published localization of GFP-Mps1 in hypoxic syncytial embryos using our CO_2_ apparatus (Figure 5A). We then used those same conditions to examine live embryos expressing GFP-Polo and His2AvD-tDimer proteins. Similar to GFP-Mps1, GFP-Polo was found to accumulate in response to hypoxia (Movie S3). However, the pattern of localization was different; while GFP-Mps1 was enriched exclusively at mitotic kinetochores, GFP-Polo was enriched at both kinetochores and centrosomes (Figure 5B). This also shows that the meiotic and mitotic localization of these proteins differ, as in embryos they localize in different patterns, while in oocytes Mps1 and Polo were found together at both the filaments and meiotic kinetochores [20].

## Discussion

The experimental data presented here demonstrate that Mps1 and Polo localization to the ooplasmic filaments is a transient response to hypoxic conditions, with the GFP-labeled proteins loading on and off of static filaments on a time scale of approximately 10 minutes. This is not an artifact caused by the GFP tag, as filaments were first identified by antibodies against the native proteins [20]. This dependency on hypoxia explains the observations in our previous study of Mps1 and Polo localization to the filaments, as groups of flies were anesthetized with CO_2_ prior to dissection. This was done on a time scale (no more than 15 minutes from initial CO_2_ exposure to addition of the fixation buffer) that was very close to the filament transition time. This was a serendipitous coincidence; if the transition time was on the order of 1 minute, the proteins would have had time to dissociate from the filaments between removal from the CO_2_ plate and fixation, while if the transition time was on the order of 20 minutes or longer, they would not have had time to complete their initial association with the filaments prior to fixation. In either situation, we would have never observed the filaments. This type of localization also explains a number of other features of the filaments; for instance, some fixed oocytes have filaments with higher or lower contrast to the ooplasmic background, which can be explained by those oocytes having a higher or lower proportion of the available protein sequestered to the filaments at the moment of fixation.

The structural proteins underlying the filaments also appear to remain during normoxic conditions and only become identifiable when the Mps1 and Polo proteins are localized to them. This conclusion is supported by three observations. First, during the initial appearance of filaments during NEB, they appear to be polymerizing linearly, in a wave that propagates from the posterior end of the oocyte (Figure 1) [20]. If the filaments were not forming at NEB and were instead forming at the point hypoxia was applied, then we would expect to find filaments that had been fixed in mid-growth during other stages of oogenesis. However, filaments that appear to still be in the process of growing (based on their short and relatively uniform lengths) have never been observed in oocytes that have clearly progressed into prometaphase. Second, during localization of GFP-Mps1 or GFP-Polo to filaments in live oocytes, the filaments light up and fade out all along their lengths instead of growing from one end (Movie S1). Third, after restoration of normoxia, a subsequent return to hypoxia causes the filaments to reappear in the same places (Figure 2E). This pattern is consistent with the sequestration of proteins to an underlying filament that remains present through normoxia. Taken together these observations also appear to indicate that the underlying filaments have fully formed prior to the initial entry into hypoxia. An analogy that closely reflects this type of structure would be a flock of birds landing on a thin wire. While the birds are in flight, the wire is still present but cannot be seen. However, once the birds come to roost, the location of the wire is easily inferred, and when the flock takes flight again the wire is still present.

The EM images also reveal that the localization of Mps1 to filaments is in well-structured rows that only occupy a small proportion of the available surface of the much larger filaments. However, what proportion of the electron-dense structure highlighted by immuno-EM is the underlying structural proteins versus other proteins that are transiently sequestered to it is not yet known. The triple-helical appearance of the immunogold label, and the sensitivity of the Mps1 and Polo localization to collagenase, are consistent with the underlying protein scaffold being a collagen-like protein. However, we note that the genome of *Drosophila* only has three identified collagen genes, and entirely lacks a canonical Type I collagen [32], which is the type known to form long linear triple-helical filaments. We have been able to test *vkg* and *CG42543* (one with Vkg-GFP, the other with anti-Mps1 antibody in a *CG42543^f07253^* homozygote, both data not shown) neither of which appeared to affect the filaments. While it is possible that Dcg1 is part of the filament backbone, the typical net-like structure of Type IV collagens [38] does not appear consistent with the filaments highlighted by Mps1 and Polo. Furthermore, there are reasons to expect that there are other collagen-like genes in the genome that have not been annotated. One study [39] screened a *Drosophila* library for clones that hybridized to a chicken collagen sequence. They then localized those clones through *in situ* hybridization, and identified 10 putative collagen-like loci across the fly genome. Two of these corresponded to *Dcg1* and *vkg*, but the remaining eight loci identified in that study have never been cloned. Furthermore, *CG42543* did not map to any of the loci identified by hybridization (www.flybase.org). Therefore, it is reasonable to expect that there are a non-trivial number of potential collagen-like genes that could be examined in an exhaustive candidate-based approach.

We also cannot rule out the possibility that collagenase is cleaving one or more proteins that are required for the localization of Mps1 to the filament, rather than the structural proteins in the filament itself. The recognition site for the collagenase enzyme is Pro-X-Gly-Pro, where X is usually a neutral residue [35]. While eGFP, Mps1 or Polo do not contain any such recognition sites, searching all annotated protein sequences in the *D. melanogaster* genome for that sequence identifies 915 genes that contain one or more potential cleavage sites, including the three annotated collagen genes mentioned above. One possible approach to identifying the structural components of the filaments would be to immunoprecipitate Mps1 or Polo under hypoxic and normoxic conditions and use proteomics to identify those proteins that only precipitate under hypoxic conditions. We have attempted this approach, and while we have been successful in getting Mps1 and Polo to co-IP under hypoxic conditions (WDG, NMS and RSH, unpublished data), attempts to wash the precipitates sufficiently for proteomic analysis were unsuccessful. We believe that this is either due to the transient nature of the association of Mps1/Polo with the filaments being too tenuous to pull down the very large filament backbones, or the association between the targeted protein and the backbone becoming released in the washing buffer. Future attempts that chemically crosslink the proteins to the filaments may prove successful.

We also report that hypoxia induces a change in the localization of *Drosophila* Polo during mitosis. There have been recent studies showing a role for Polo-like kinases in the hypoxic response in other organisms; a microarray study in rats identified Polo-like kinases being upregulated in a hypoxic tumor model [40], while in mice Plk3 was found to act as a regulator of hypoxia-inducible factor-1α under hypoxic conditions [41]. This suggests that the relocalization of Polo in response to hypoxia may be evolutionarily conserved, and opens up the powerful *Drosophila* system for studying the role of Polo in hypoxia. Interestingly, while GFP-Mps1 only localizes to the mitotic kinetochores under hypoxia, and is not normally detectable on chromosomes [21] (Figure 5A), our data show that during mitosis GFP-Polo is localized to the spindle, centrosomes and kinetochores during the normoxic cell cycle and that the localization to kinetochores and centrosomes becomes more intense under hypoxia (Figure 5B). The patterns of Polo and Mps1 localization in mitosis are different than in female meiosis, where the two proteins appear to localize together at kinetochores and filaments.

What is the function that requires the sequestration of these proteins to filaments? The cell must expend considerable energy assembling these filaments at NEB, in addition to the costs incurred in transiently sequestering proteins to them. As localization to the filaments has not been observed in mitotic cells and the filaments appear to be disassembled soon after fertilization [20], [21], they do not seem to be needed during mitosis. What is so different about the oocyte that would require constructing these extensive structures during prometaphase? While speculative, one possibility is that the hypoxic signal may be needed throughout the ooplasm, but that signal (analogously to other intracellular signals such as the spindle assembly checkpoint signal generated at kinetochores that are not under tension [17], [42]) can only be generated by proteins at kinetochores. Generating a signal at kinetochores and distributing it throughout the very large oocyte (either by diffusion or active transport) may be impractical for a signal that needs to propagate on a short time scale. Perhaps the sequestration of these proteins to filaments brings them together in the proper orientation to allow the generation of the hypoxic signal. In other words, the filaments could be serving as surrogate kinetochores, enabling the generation of the signal simultaneously at many places throughout the oocyte. This hypothesis would suggest the filaments form at NEB because this mechanism is not needed in the presence of an intact nuclear envelope, while the filaments can be disassembled after fertilization because the rapidly increasing number of mitotic nuclei would make such surrogate kinetochores unnecessary. Consistent with this interpretation, the very similar-appearing filaments identified in *C. elegans* during prometaphase I in female meiosis also localize several outer kinetochore proteins [23], although we are unaware of any studies that have examined whether the localization of these proteins to filaments in nematodes is sensitive to hypoxia. Alternatively, the function of these filaments may be to sequester these proteins, either to attenuate their activity or to protect them from depletion during hypoxia. Answering this question will ultimately require the identification of the structural proteins that make up the filaments, so that the consequences of knocking them out can be studied.

Finally, this study should serve as a cautionary tale for the use of fixed imaging to study dynamic processes. Even routine laboratory procedures (such as CO_2_ anesthesia) can inadvertently introduce dramatic differences in the system under study. While it is tempting to dismiss such things as being incompletely penetrant or just variable phenotypes, careful control of the experimental conditions can lead to novel discoveries. It also emphasizes the advantages of doing live imaging in parallel with fixed imaging. Not only did we benefit from the stark differences between the fixed and live samples, we would certainly never have been able to demonstrate that the GFP filaments are reappearing in the same places without live imaging data.

## Materials and Methods

### Fly Stocks

All flies were aged for 3–5 days with males and fresh yeast paste prior to imaging. For fixed and live oocytes, flies were collected from homozygous stocks containing a *GFP-polo* transgene inserted on the *X* [25] or two independent insertions of the same *GFP-mps1* transgene on chromosome *2* [16]. This copy number difference is one of the reasons why fluorescence of GFP-Polo is inherently fainter than GFP-Mps1. For imaging of mitosis, virgin females from either the *GFP-mps1* or *GFP-polo* stocks were crossed to *his2AvD-tDimer/CyO* males [37], and male and female progeny carrying both transgenes were collected and aged. Flies were then transferred to egg collection cages with grape juice agar plates and yeast for one hour. Embryos were then collected and dechorionated with 50% bleach prior to imaging in halocarbon oil as described for live oocytes below.

### Oocyte Incubation

Several different conditions were used to incubate oocytes prior to fixation. **Standard Incubation:** The condition used in [20] was to anesthetize 10–15 females on a CO_2_ plate, hand dissect females into 1x Modified Robb's Media [44], with ovaries being transferred to a tube containing Robb's as they were removed. Once all dissections were done, buffer was removed and fixative was added as described below. A timer was used to ensure that no more than 15 minutes elapsed from dissecting the first female to application of fixative. With this protocol a mix of oocytes is obtained, some with and some without Mps1 or Polo localizing to filaments, due to some females being exposed to CO_2_ for longer periods of time. **Normoxic Incubation:** Three females at a time were anesthetized and dissected as quickly as possible followed by immediate fixation. When more than three females were required, several batches of three females were fixed and then pooled after fixation. No oocytes were observed to have filaments under this treatment, due to fixation occurring so soon after initial CO_2_ exposure that the Mps1 or Polo has not had time to be sequestered to filaments, which in live imaging requires 7–8 minutes for filaments to initially become visible. **Hypoxic Incubation:** 10–15 females were dissected as per the standard conditions, then the tube containing Robb's was closed and oocytes were incubated for 30 minutes prior to fixation. This treatment resulted in 100% of post-NEB oocytes having visible Mps1- or Polo-associated filaments. **Sodium Azide Incubation:** Three females were quickly anesthetized and dissected, with ovaries immediately transferred to open eppendorf tubes containing 0.7% NaCl, with or without 0.03% sodium azide, for 10 minutes prior to fixation. NaCl was used in place of Robb's to avoid potential reactivity between the azide and the components of the Robb's media. Sodium azide inhibits mitochondrial respiration and can trigger the hypoxic response in *Drosophila* embryos [21]. **Long Duration Live Imaging:** 1–3 Females were dissected into halocarbon oil 700 (Sigma), and individual stage 13 oocytes were transferred to fresh halocarbon oil on a well slide (made by placing a square border made of electrical tape on a no. 1 1/2 cover slip) using a micro-hook. Oocytes were then positioned, injected (if necessary) using standard microinjection procedures, with the needle inserted halfway between the midline and posterior end of the oocyte. Our injection apparatus did not allow quantification of injected volume; successful injection was monitored by the clearing of ooplasm around the needle tip. After injections were completed, the slide was covered with a piece of YSI membrane. Once the slide was on the microscope, a chamber was placed over the stage (PeCon Incubator XL LSM S for the Zeiss; a cardboard box for the Deltavision) and oocytes were made hypoxic by pumping gas (either N_2_ or CO_2_) into the chamber. To restore normoxia the flow of gas was turned off, without disturbing the chamber (to avoid moving the stage). For heat shock experiments the PeCon's temperature controlled stage was used to maintain a stage temperature of 37°C.

### Oocyte Fixation

Oocytes were fixed in 1.3 ml of a 1∶1 mixture of 16% paraformaldehyde (Ted Pella) and 2x Fix Buffer (200 mM potassium cacodylate, 200 mM sucrose, 200 mM sodium acetate, and 20 mM EGTA). Oocytes were fixed for 4 minutes with rocking on a nutator, followed by washing in PBST (PBS plus 0.1% Triton-X100). Dechorionation and antibody hybridization of fixed oocytes were performed as previously reported [20], but for fixed preps with only GFP, ovarioles were separated by rapidly pipetting up and down with a 1000 µl pipette, washed again in PBST, stained in 495 µl PBS plus 5 µl of 10 µg/ml DAPI for 6 minutes, then washed 5 times in PBST prior to mounting in Slowfade Gold.

### Fluorescent Microscopy

Fixed oocytes (Figures 1 and S1) were imaged on a Deltavision deconvolution microscope using the SoftWoRx software package (Applied Precision). Live imaging was done on either a Deltavision (Figures 2, 4, and Movie S1) or a Zeiss LSM 510 scanning laser confocal microscope (Figure 5 and Movies S2, S3) using the AxioVision software package. Images and movies acquired on the Deltavision were deconvolved prior to maximum-intensity stack projection, except for the 10x image in Figure 1A.

### Immuno-Electron Microscopy

Ovaries from five GFP-Mps1 females were dissected into Robb's media and incubated in a closed eppendorf tube with 1.5 ml Robb's media plus 65 µM colchicine for 30 minutes. Colchicine has a limited effect on the number or length of the filaments [20] but does appear to provide higher contrast GFP images. Then buffer was removed and oocytes were fixed by addition of fixation buffer as described above. After 4 minutes, fixative was removed and oocytes were washed with PBS. (No detergent was used; to prevent sticking, all plasticware was blocked by pipetting PBS containing 4% BSA, then air dried prior to use.) Ovarioles were then separated as above (using PBS instead of PBST), and individual stage 13–14 oocytes with visible dorsal appendages were selected on a dissecting microscope and transferred to a drop of PBS on a cover slip well slide on the Deltavision. Oocytes that displayed robust GFP filaments were then postfixed in 2% paraformaldehyde and 0.01% glutaraldehyde in 0.1 M PBS, then infused with 2.3 M sucrose and polyvinylpyrrolidone (PVP) in 0.1 M PBS overnight at 4°C. Individual oocytes were frozen on ultracryotome stubs in liquid nitrogen and stored in liquid nitrogen until use. Ultrathin sections (50–70 nm) were cut using a Leica EM UC6 ultramicrotome with a FC6 cryo-attachment, lifted on a small drop of 2.3 M sucrose and mounted on Formvar-coated copper grids. Sections were washed three times with PBS, then three times with PBS containing 0.5% bovine serum albumin and 0.15% glycine followed by a 60 min incubation with 1% normal goat serum. Sections were labeled with mouse anti-GFP monoclonal antibody (3E6, Invitrogen, used at 1∶50) at room temperature for 1 hr, washed, then labeled with goat anti-mouse IgG 10 nm colloidal gold conjugate antibody (G7777, Sigma, used at 1∶25) at room temperature for 1 hr. Sections were post-stained in 2% neutral uranyl acetate for 7 min, washed three times in ddH_2_O, stained 2 min in 4% uranyl acetate, then embedded in 1% methyl cellulose. Labeling was observed on a FEI electron microscope at 80 kV.

### Image Preparation

Figure 1B is a composite image from 5 separate 1024×1024, 10 µm Z-stacks (Δz = 0.2 µm). Images were deconvolved, projected and manually aligned as layers in Photoshop, using GFP filaments and follicle cell nuclei in overlapping regions to guide alignment. The rectangular region was then copied, individual layers were composited using layer masks, adjusted with the Layers function, and then merged; the slight variation in brightness across the background is due to slight differences across the five original images. The region around the oocyte nucleus is a 2 µm subset of the full Z stack, to make the nuclear envelope easier to see. No GFP filaments were observed in the full Z stack for this region of the image. For Figure 2E, the before-and-after images (2B and 2D, respectively) were placed as Photoshop layers in the same file. Because the cell and stage had drifted slightly over the intervening 30 minutes, the features in the center of each image were manually aligned. The Before layer was put on top, colored green (by setting the red and blue RGB channels to black) and that layer's blending mode was set to Screen. The After layer was colored red (by setting the green and blue RGB channels to black). Both layers were then adjusted using the Levels function to emphasize the filaments, minimize background, and to make both layers appear approximately equally bright. Composite images in Figure 4 were collected using the SoftWoRx Panels function and merged with the Stitch function. For Figure 5, screen captures from the indicated time points in the movie were taken and then merged to a single layer prior to the application of the Auto Contrast function.

### Helical Modeling

Consider a cylinder of radius *r*, with the center of the cylinder along the X axis. Call the length for a helix to complete one revolution *L*, such that the position along the X axis at a given angle is θ (*L*/2π). Therefore, the XYZ coordinates of helical points along the surface of the cylinder can be calculated as a function of the helical angle θ, such that X(θ) = θ (L/2π), Y(θ) = −*r* cos(θ), and Z(θ) = *r* sin(θ). The negative sign on Y(θ) reverses the handedness of the helix. Using Photoshop to measure pixel distances in EM images and comparing them to the scale bar, we determined that *r*≈75 nm and L≈270 nm. This formula was repeated for the second and third helices, rotating the helices around the cylinder by adding 2π/3 and 4π/3 to the value of θ in the Y(θ) and Z(θ) functions. To approximate a bend in the cylinder, a function B(θ) was added to the value of Z(θ); through trial and error we found that a curved cylinder with B(θ) = sin (θ/4) 325 nm appeared to approximate the EM data. The formulas were calculated and plotted in Excel. Figure 3C plots the XYZ coordinates for a triple helix calculated at 200 steps from 0≤θ≤4π (two complete rotations), with simulated sections through the cylinder made by using Excel's IF() function. A 70 nm cross section was simulated by only plotting those points within 0≤X≤70, while a 70 nm transverse section was simulated by only plotting those points within 255≤Z≤325.

### Sequence Analysis

The chado_proteins.xml file containing all annotated protein sequences was downloaded from Flybase (www.flybase.org) on May 27, 2009. The XML file was then processed with Perl scripts to eliminate non*-melanogaster* sequences, to group multiple transcripts of the same gene, and to evaluate the regular expression /P[AGVLIPFMWCNQSTWY]GP/ which identified sequences with putative collagenase restriction sites.

## Supporting Information

Figure S1 Sodium azide treatment can induce filament formation. GFP-Mps1 oocytes were incubated under different conditions prior to fixation, and stage 13 oocytes were examined for localization to filaments. Oocytes fixed while normoxic (where fixation occurs so quickly after initial CO2 exposure that the GFP-Mps1 has not had time to be sequestered to the filaments) never showed localization to filaments. The exposure of oocytes to sodium azide for 10 minutes causes a significant increase in the number of oocytes that show GFP localization to filaments, when compared to incubation alone (Fisher's Exact Test, P<0.0001). Sodium azide inhibits mitochondrial respiration and can induce the hypoxic response in mitotic Drosophila cells [21]. The exceptional oocytes in each treatment are also consistent with localization being controlled by hypoxia, as all three sodium azide-treated oocytes without filaments had fully mature dorsal appendages, while the single control oocyte with filaments was an early stage 13 oocyte with poorly developed dorsal appendages. The dorsal appendages are gill-like structures used by the oocyte for respiration [43]. Therefore, having poorly developed dorsal appendages would be expected to predispose the oocyte to hypoxia due to incubation alone, while mature dorsal appendages would be expected to provide better baseline oxygenation, which would be expected to make sodium azide take longer to trigger the hypoxic response. Finally, all oocytes fixed while hypoxic (after a 30 minute incubation in a sealed eppendorf tube) had GFP-Mps1 localized to filaments.
(0.80 MB TIF)

Movie S1 A live GFP-Mps1 oocyte shows GFP localization to filaments in response to hypoxia. This stage 13 GFP-Mps1 oocyte was imaged prior to application of CO_2_ (Fig. 2A), and then hypoxia was induced by turning on the flow of CO_2_. The first filaments became visible after approximately eight minutes of hypoxia. Then a 10 µm image stack (Δz = 0.2 µm) was acquired (Fig. 2B). Live imaging began with 2 µm stacks (Δz = 0.4 µm) acquired every 90 seconds. After the first time point (t = 0:00), the flow of gas was turned off, restoring normoxia. This caused the GFP filaments to disperse back into the ooplasm, with no filaments visible by t = 13:30 (Fig. 2C). The gas was turned back on after this time point, and the filaments first became visible again approximately 7:30 later (t = 21:00), and then became progressively brighter through the end of filming at t = 30:00. With the CO_2_ still on, a 10 µm image stack (Δz = 0.2 µm) was acquired (Fig. 2D). All times are rounded up to the nearest second.
(5.83 MB MOV)

Movie S2 Heat shock does not induce GFP-Polo localization to filaments. A stage 13 GFP-Polo oocyte was heated to 37°C 18 minutes before live imaging commenced. This did not induce GFP localization to filaments. Seven other oocytes on the same slide were also monitored but not live-imaged; all exhibited similar localization before, during and after imaging as the oocyte shown. CO_2_ was turned on after the first frame, and the filaments appeared shortly thereafter, indicating the cell was still capable of sequestering GFP-Polo to filaments at the elevated temperature. After the eighth time point (5:15), filming was paused while the other seven oocytes were examined. After filming resumed (12:22), the CO_2_ was turned off, and the GFP-Polo dispersed back into the ooplasm.
(1.89 MB MOV)

Movie S3 GFP-Polo localization to mitotic kinetochores and centrosomes increases in response to hypoxia. A syncytial embryo with GFP-Polo and red fluorescently labeled histone (His2AvD-tDimer [37]) completes one cycle of mitosis; a subset of this oocyte is shown in Figure 5B. CO_2_ gas was turned on after 6:15, and the cell arrested in metaphase of that cell cycle. After 11:30, the gas was turned off, and the embryo resumed mitotic cycling thereafter, reaching metaphase of the next mitotic cycle approximately 25 minutes later. The second cell cycle in this video is prolonged because the onset of hypoxia is much faster than the return to normoxia, as CO_2_ is quickly pumped into the gas chamber. However, removal of the chamber caused a loss of microscope focus and stage positioning, so to be able to best monitor the oocyte throughout imaging we simply opened the cover to allow ambient air to return by diffusion.
(8.70 MB MOV)
