# Supplementary figures and images for: Hypoxia Transiently Sequesters Mps1 and Polo to Collagenase-Sensitive Filaments in Drosophila Prometaphase Oocytes
Source: PLoS One. 2009 Oct 22;4(10):e7544. doi: 10.1371/journal.pone.0007544 (PMC2761730; doi:10.1371/journal.pone.0007544)

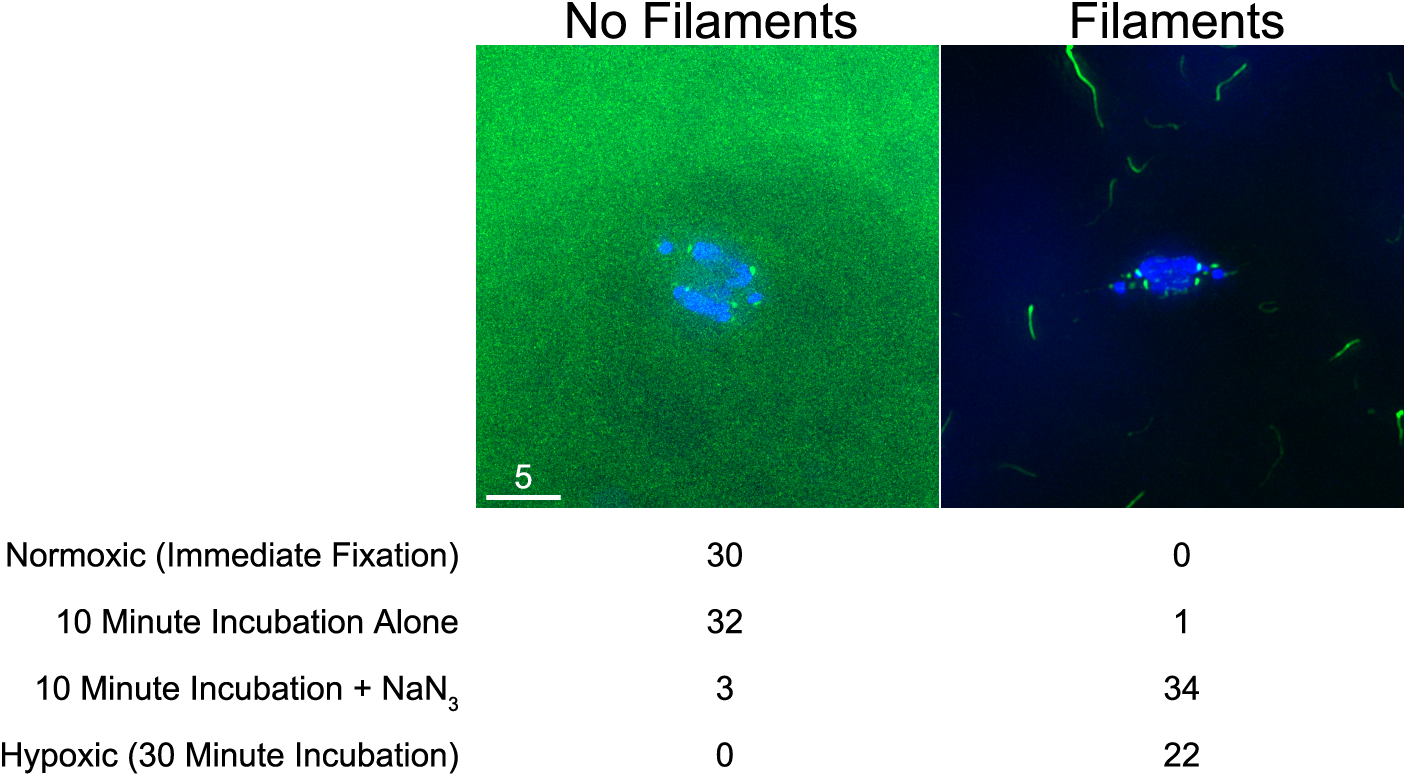

Supplement: Figure S1 — Sodium azide treatment can induce filament formation. GFP-Mps1 oocytes were incubated under different conditions prior to fixation, and stage 13 oocytes were examined for localization to filaments. Oocytes fixed while normoxic (where fixation occurs so quickly after initial CO2 exposure that the GFP-Mps1 has not had time to be sequestered to the filaments) never showed localization to filaments. The exposure of oocytes to sodium azide for 10 minutes causes a significant increase in the number of oocytes that show GFP localization to filaments, when compared to incubation alone (Fisher's Exact Test, P<0.0001). Sodium azide inhibits mitochondrial respiration and can induce the hypoxic response in mitotic Drosophila cells [21]. The exceptional oocytes in each treatment are also consistent with localization being controlled by hypoxia, as all three sodium azide-treated oocytes without filaments had fully mature dorsal appendages, while the single control oocyte with filaments was an early stage 13 oocyte with poorly developed dorsal appendages. The dorsal appendages are gill-like structures used by the oocyte for respiration [43]. Therefore, having poorly developed dorsal appendages would be expected to predispose the oocyte to hypoxia due to incubation alone, while mature dorsal appendages would be expected to provide better baseline oxygenation, which would be expected to make sodium azide take longer to trigger the hypoxic response. Finally, all oocytes fixed while hypoxic (after a 30 minute incubation in a sealed eppendorf tube) had GFP-Mps1 localized to filaments. (0.80 MB TIF) [file pone.0007544.s001.tif]
